# COVID-19-Induced Ischemic Infarcts With Normal Coagulation Profile and Excellent Outcome: A Case Report With Highlights on the Thrombotic Diathesis, Recent Transcranial Doppler Findings, and Neuropathology Update

**DOI:** 10.7759/cureus.12529

**Published:** 2021-01-06

**Authors:** Hassan Kesserwani

**Affiliations:** 1 Neurology, Flowers Medical Group, Dothan, USA

**Keywords:** stroke, covid-19, covid coagulopathy

## Abstract

We present the case of a previously healthy 41-year-old man right-handed man diagnosed with coronavirus disease 2019 (COVID-19) who developed multiple cortical ischemic infarcts. Our patient developed mild neurologic deficits despite the total volume of cerebral ischemic lesions, presumably due to involvement of non-eloquent cortex, the integrity of the collateral circulation due to his youth and good health, and mild COVID-19 disease without any significant pulmonary involvement. We discuss the coagulopathy and direct vascular effects, if any, of COVID-19 disease and compare it to other agents such as the filovirus, ad Ebola. We also outline the recent intriguing findings of vasodilation of the pulmonary vessels and the potential shunting of micro-emboli into the brain, which may explain the formation of embolic ischemic infarcts in even mild disease. Lastly, we discuss the neuropathology of COVID-19 in patients that who have succumbed to the disease and note the striking lack of direct involvement of the cerebral blood vessels.

## Introduction

Acute ischemic stroke (AIS) is a complication of coronavirus disease 2019 (COVID-19) infection. A review of 39 studies revealed an incidence of AIS of 1.2%, that is, 54 out of 4,466 patients, with a mean age of 63.4 years. Laboratory studies revealed elevated domain dimer (D-dimer) and fibrinogen levels. Anti-phospholipid antibodies were detected in a significant number of cases, close to 17%. Neuroimaging studies revealed large vessel thrombosis, embolism, or vascular stenosis in 62.1% of patients. Multiple infarcts were found in 26.2% of patients, with a mortality rate as high as 38% [1]. Very high D-dimer levels and elevated fibrin degradation products (FDPs) in COVID-19 infections are also associated with a high mortality rate following ischemic strokes [2].

Disseminated intravascular coagulation (DIC) is characterized by activation of the coagulation system, leading to thrombi in the micro-circulation. Tissue factor (TF), a transmembrane protein found on sub-endothelial cells, activates the extrinsic coagulation pathway, which triggers thrombin generation and fibrin deposition. A positive D-dimer test is a marker of DIC, indicating activation of the clotting and fibrinolytic systems. This may lead to both intravascular thrombosis and a hemorrhagic tendency.

A prolonged activated partial thromboplastic time (aPTT) may indicate a clotting factor deficiency or the presence of an inhibitor of coagulation such as an antibody to factor VIII or a lupus anticoagulant. A lupus anticoagulant is associated with an increased risk of thrombosis. Around 91% of patients with severe acute respiratory syndrome coronavirus 2 (SARS-CoV-2) were positive for a lupus anti-coagulant, and many had factor XII deficiency [3].

Coagulopathy is seen in the sickest patients. The coagulation profile of COVID-19-associated coagulopathy suggests a thrombotic diathesis. As noted above, this is reflected by an increase in D‐dimer levels and FDPs, which is a marker of fibrin formation as seen with DIC. This is in contrast with bacterial sepsis-associated coagulopathy where a prolongation of prothrombin time (PT), aPTT, a decrease in antithrombin activity, and thrombocytopenia occur more commonly. Lymphopenia is a consistent finding in COVID-19 infection. Tissue factor is also activated and triggers the extrinsic coagulation cascade. Thrombus formation in the microvascular circulation leads to organ ischemia. This is in contrast with the coagulopathy of Ebola infection, which is profiled marked by thrombocytopenia, fibrin deposition, increased FDPs, and prolongation of PT and aPTT, which lead to a hemorrhagic diathesis, as in bacterial sepsis [4-7].

A previously healthy 41-year-old healthy right-handed male diagnosed with COVID-19 who developed multiple cortical ischemic infarcts was evaluated. What is surprising and intriguing about our case is the fact that our patient did not develop a frank coagulopathy. He had normal D-dimer levels, PT, and aPTT, and a negative screen for the anti-phospholipid antibody/lupus anti-coagulant syndrome. Furthermore, he did not develop any respiratory distress. There was no obvious embolic source with transesophageal echocardiography (TEE) or cardiac event monitoring. Based on his presentation, we were prompted to review the data on coagulopathy triggered by viruses, neuropathology, and recent transcranial Doppler (TCD) findings in COVID-19-induced ischemic strokes in order to better understand the clinical profile of our patient.

## Case presentation

We describe the case of a healthy 41-year-old male who developed constitutional symptoms of low back pain, fatigue, and fevers rising to 101.3 degrees Fahrenheit for seven days. By the eighth day, he developed a loss of taste and mild shortness of breath at rest. His initial COVID-19 identification test with a nasopharyngeal swab and identification of viral ribonucleic acid (RNA) with a reverse transcriptase (RT) test was negative. He was given a course of antibiotics and steroids. All his symptoms resolved except for mild shortness of breath. On the 20th day, he was standing in the garage holding a piece of metal, which inadvertently dropped to the floor. His son noted that his speech was slurred and that he was unable to open the tap to wash his hands. He was rushed to the hospital where he was briefly unable to comprehend the questions on the forms.

His past medical history was significant for mild hyperlipidemia treated successfully with atorvastatin 20 mg daily. There was no family history of pre-mature coronary artery disease or ischemic strokes.

On examination, his blood pressure was 132/68 mmHg, pulse was 82 beats per minute, temperature was 98.0 degrees Fahrenheit, a saturated oxygen SaO2 level was 96%, height was 183 centimeters, weight was 99.7 kilograms, and BMI was 29.77. The patient was comfortable and in no respiratory distress. Precordial auscultation revealed no murmurs, and carotid auscultation revealed no bruits. Breath sounds were audible throughout both lungs.

His station, gait tandem, and cadence were normal. He was cognitively slow with some delay in answering questions, but he was able to repeat and to name objects, and he had a good understanding of conversation. No dysarthria was noted. Cranial nerve examination was normal. Specifically, there was no evidence of a visual field cut or visual field neglect with confrontation. Motor testing showed no evidence of a pronator drift, and sequence motion of the fingers was symmetric and normal. Muscle group testing showed no weakness. No sensory extinction was noted with double simultaneous touch on both arms. Graphesthesia and stereognosis were preserved in his hands. No dysmetria or intention tremor was noted in the upper or lower extremities. In summary, his neurologic examination was only significant for mild cognitive slowing.

A repeat COVID-19 nasopharyngeal swab polymerase chain reaction (PCR) test was positive. Pertinent positive laboratory testing included a mild leukocytosis of 11.8 thousand per microliter (4-11 thousand per microliter of blood) with a differential of neutrophils of 85.6% (40-75%) and lymphocytes of 11.3% (20-50%). The rest of the white cell differential was within normal limits. There was no evidence of an anemia or a thrombocytopenia. Renal function and hepatic liver enzymes were normal. His prothrombin PT was normal. aPTT was normal and so was the screen for the anti-phospholipid antibodies/lupus anti-coagulant syndrome. Plasma D-dimer quantitative level was 218 ng/mL (normal: <230 ng/mL). Thyroid-stimulating hormone (TSH) and hemoglobin A1C (HgbA1c) were normal. His fasting lipid profile was within normal limits. A serum fibrinogen level test was not done. Protein C and protein S were normal.

**Figure 1:**
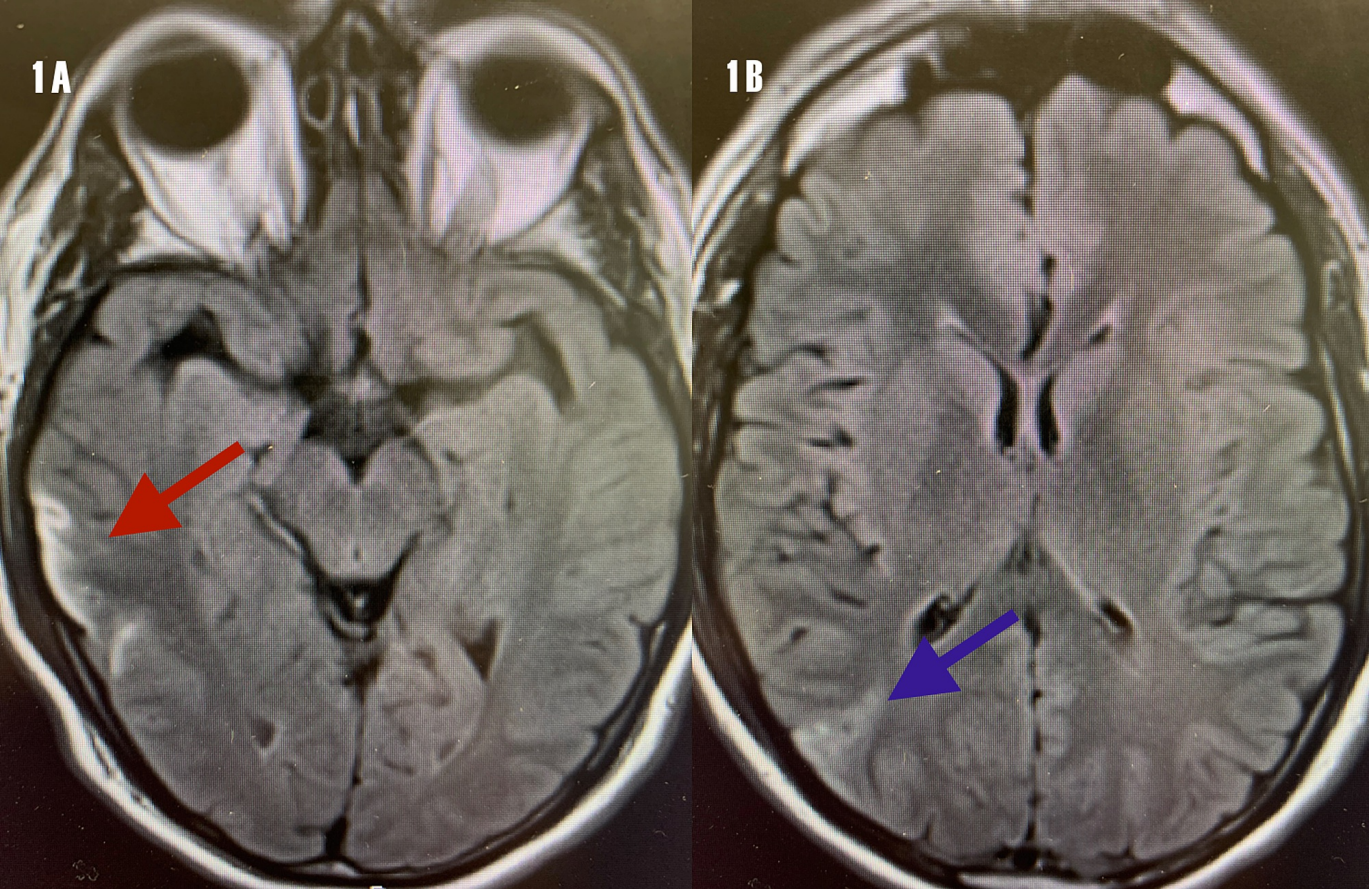
FLAIR MRI. (A) Right cortical temporal ischemic infarct (red arrow). (B) Right posterior parietal cortical ischemic infarct (blue arrow). FLAIR MRI, fluid-attenuated inversion recovery magnetic resonance imaging

A magnetic resonance image (MRI) of the brain revealed cortical ischemic infarcts on diffusion-weighted images (DWIs) in the right temporal and right parieto-occipital cortices. However, due to the under-penetrated quality of images, we submit the fluid-attenuated inversion recovery (FLAIR) MRI images, which demonstrate the cortical infarcts (Figure 1).

**Figure 2:**
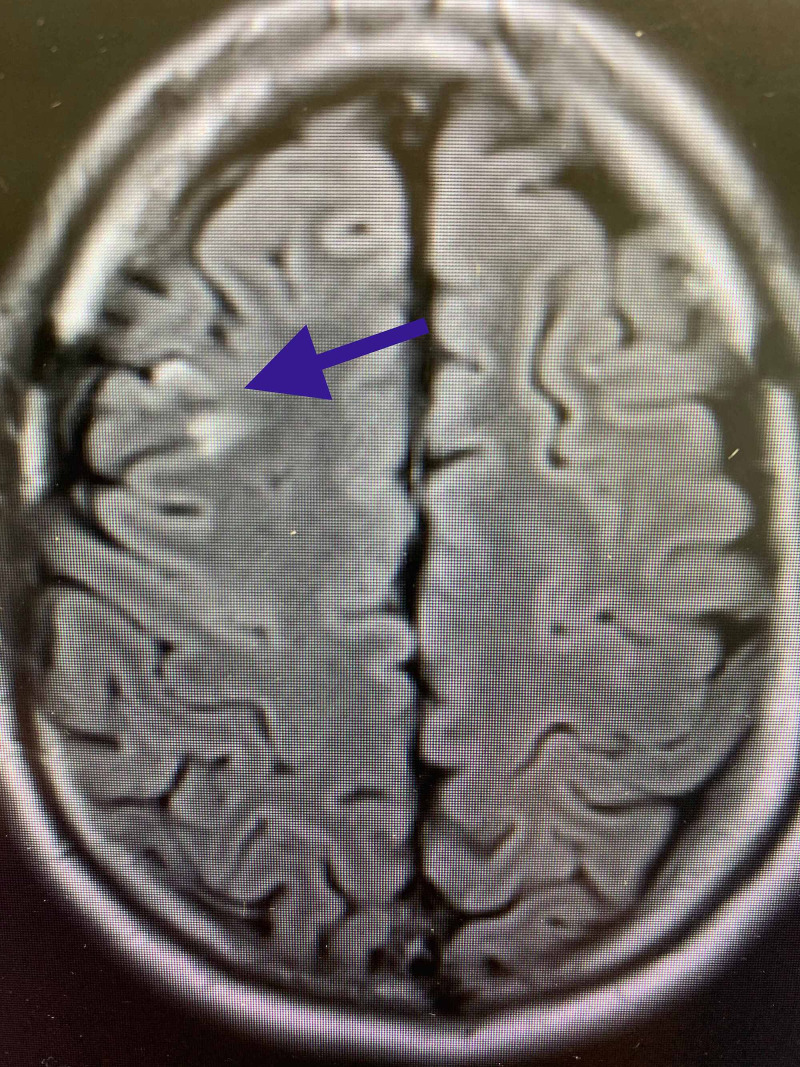
FLAIR MRI: right frontal cortical ischemic infarct (blue arrow). FLAIR MRI, fluid-attenuated inversion recovery magnetic resonance imaging

A right frontal cortical infarct is also noted on DWI images, which was replicated on FLAIR MRI images (Figure 2).

The patient’s chest X-ray was normal. A carotid duplex scan, magnetic resonance angiography (MRA) of the brain, and a transthoracic echocardiogram was normal. An outpatient TEE and a 30-day cardiac event monitor were normal. No cardiac source of emboli was identified. A cardiac loop recorder was implanted to rule out paroxysmal atrial fibrillation. SARS-CoV-2 immunoglobulin G (IgG) titers were detected seven weeks after initial infection.

The patient was treated with oral dexamethasone 6 mg daily, 81 mg aspirin daily, and subcutaneous enoxaparin at a dose of 40 mg twice daily. Due to his minimal deficits and remarkably fast recovery, the patient was discharged home after 48 hours on apixaban 5 mg twice daily. The patient was switched to 81 mg aspirin daily two weeks after discharge. The results of the cardiac loop monitor should provide further information over the next two to three years. How long the patient should have stayed on full anti-coagulation should be determined by the continually evolving data on COVID-19-induced ischemic infarcts anecdotal case series and eventually meta-analytic data. A double-blind controlled trial would be ideal. However, in the absence of a cardiac source of emboli and a normal lupus anticoagulant/anti-phospholipid antibody profile, full anti-coagulation was not justified.

## Discussion

We will take a brief detour and explore the mechanisms of vascular injury of various infectious agents, such as the varicella-zoster virus (VZV), Rocky Mountain spotted fever (RMSV), and Ebola, in order to set the stage for COVID-19-induced vascular phenomena. VZV antigens and neutrophils invade the adventitia in VZV vasculopathy. There is also thickening of the tunica intima and inflammation of the vaso vasorum. Late VZV vasculopathy is characterized by viral antigen without inflammation in the tunica media of blood vessels [8]. *Rickettsia rickettsii*, the causative agent of RMSF, damages endothelial cells, leading to thrombosis, hemorrhage, and vasculitis. Ebola virus infection of endothelial cells does not lead to an endothelial cytopathy [9]. Therefore, viral infections can induce vascular thrombo-inflammation through various mechanisms.

As a prelude to our discussion, we define the innate immune system as an "a priori " line of defense with macrophages, neutrophils, and natural killer cells at the forefront, with elaboration of interferons. The adaptive immune system is a “tabula rasa” system and involves an anamnestic response with the active participation of bursa of Fabricius cells (B-cells), thymic cells (T-cells), and the major histocompatibility antigen system. The latter line of defense involves the elaboration of specific antibodies. Both defense mechanisms are involved in the immune response to COVID-19.

We will next compare the immune response of the Ebola virus and the COVID 19 virus. DIC is a manifestation of Ebola virus infection, which has similarities to bacterial septic shock. Both can lead to lymphocyte depletion and a rise in inflammatory cytokines, and both activate the coagulation cascade, leading to thrombocytopenia, prolonged PT and aPTT, low serum fibrinogen, and low platelet count. However, the Ebola virus induces a hemorrhagic rather than a thrombotic state [9]. Low protein C levels are also found in the majority of patients with sepsis and are associated with an increased risk of death [10]. In COVID-19 pneumonia, there is damage to lung cells and an invasion of endothelial cells by the COVID-19 virus, which triggers an immune inflammatory response, activating the innate immune system. There is also elaboration of cytokines such as interleukin- 6 (IL-6), a pro-coagulant, complement activation, and TF elaboration, activating the extrinsic factor coagulation system. The virus enters into cells through the angiotensin-converting enzyme 2 (ACE-2) receptor, which is expressed on monocytes. T-cells interact with antigen-presenting cells, initiating the adaptive immune response [11]. This thrombo-inflammation may also activate or worsen pre-existing conditions.

AIS may also be due to rupture of a carotid plaque, carotid thrombosis, or the development of atrial fibrillation induced by the inflammatory response. Invasion of endothelial cells by the COVID-19 virus with inflammation and apoptosis of endothelial cells has been shown to occur in renal, cardiac, gastrointestinal, and pulmonary tissue at autopsy, but not in cerebral tissue [12]. These remarkable findings may offer insights into the mechanisms of presumed idiopathic or cryptogenic ischemic strokes in non-COVID-19 ischemic stroke patients.

Reynolds et al. have recently explained the paradox between normal lung compliance with severe hypoxia in COVID-19 patients and respiratory failure. They deployed TCD insonation of the middle cerebral arteries after intravenous injection of agitated saline. Normally, these micro-bubbles are cleared by the lung unless there is an intrapulmonary shunt. They can also bypass the lungs if there is a patent foramen ovale. They studied 18 mechanically ventilated patients with severe COVID-19 pneumonia. None of the patients had an intracardiac shunt. Around 83% of the patients had micro-bubbles detected by TCD in the cerebral circulation [13]. A surrogate marker of oxygenation, the ratio of arterial oxygen tension-to-fraction of inhaled oxygenation (P/F ratio), was inversely related to the number of micro-bubbles, that is, the more micro-bubbles, the less the oxygenation. Finally, the number of micro-bubbles was inversely related to lung compliance, That is, the more the micro-bubbles, the stiffer the lungs. The authors hypothesize that pulmonary vasodilation may explain these findings in some patients. Furthermore, worsening lung function as measured by worse compliance, was proportional to the number of micro-bubbles, pari-passu with pulmonary vasodilation. This was also reflected by lower P/F ratios, reflecting severe hypoxemia. They pointed out that this phenomenon is akin to the hepatopulmonary syndrome, a condition characterized by pulmonary vascular vasodilatation and ventilation-perfusion mismatch, leading to hypoxia in patients with chronic liver disease [12,13]. This phenomenon of pulmonary shunting of micro-emboli is seen in only 26% of patients with classic adult respiratory distress syndrome [14].

Histopathological examination of brain specimens obtained from 18 patients who died of COVID-19 showed only hypoxic changes and no evidence of encephalitis. All the patients had a confusional state or decreased arousal from sedation. Immunohistochemical analysis revealed no cytoplasmic viral staining. Microscopic examination showed acute hypoxic injury with loss of neurons in the cerebral cortex, hippocampus, and cerebellar Purkinje cells [15].

In 62 patients who died from COVID-19, postmortem MRI of the brain was obtained early, within one day after death. Subcortical micro-bleeds and macro-bleeds bleeds were seen in two patients, cortical and subcortical edematous changes suggestive of posterior reversible encephalopathy syndrome were seen in one patient, and nonspecific deep white matter changes were seen in one patient. Asymmetric olfactory bulbs restricted to the olfactory bulb only were found in four patients. No brainstem MRI signal abnormalities were noted [16].

Von Weyhern et al. reported neuropathology on six fatal outcomes, with all patients requiring ventilatory support. Petechial bleeds, lymphocytic meningitis, encephalitis, and neuronal cell loss were noted pathological findings. The young (<65 years) and old (>65 years) demonstrated diffuse petechial hemorrhages. Both groups showed lymphocytic pan-encephalitis and meningitis. There was a conspicuous absence of endothelial cell damage. All brains examined showed perivascular and interstitial encephalitis, with neuronal cell loss and axon degeneration in the dorsal motor nuclei of the vagus nerve, trigeminal nucleus, nucleus tractus solitarius, dorsal raphe nucleus, and medial longitudinal fasciculus. Fatal cerebral bleeds were only seen in the younger group [17].

Song et al. recently reported their findings of the neuro-invasiveness of SARS-CoV-2 in cortical neurons of human organoids and mice over-expressing ACE-2 receptors. The mice had a higher mortality despite the absence of pulmonary disease [18].

Susceptibility-weighted images (SWIs) on MRI of nine patients who had suffered from moderate or severe acute respiratory distress syndrome revealed cerebral micro-bleeds (CMBs) in the corpus callosum. Other locations of micro-bleeds were the internal capsule (five out of nine cases) and the middle cerebellar peduncles (five out of nine patients). CMBs were seen in the subcortical regions in the majority of patients [19]. A case report with review of the topography of CMBs in various diseases is provided by the author of this article [20].

We believe that our patient's prognosis was excellent for several reasons. Firstly, he lacked the co-morbid conditions associated with poor outcome, such as obesity, hypertension, and diabetes. Secondly, his pulmonary disease was very mild, portending a great outcome. Thirdly, his cortical infarcts involved non-eloquent cortex, and his youth and good health may have allowed a good collateral circulation. We think that the cortical ischemic infarcts are due to thrombi from the coagulopathy of COVID-19 that are not filtered by the lungs. The study by Reynolds et al. provides potential supportive data for this last hypothesis [13]. Unfortunately, serum fibrinogen, also an acute-phase reactant, and an in-hospital TCD tests were not performed in our patient. This would have helped in solidifying our hypothesis. One may argue why the rest of the thrombophilia panel was not done, such as anti-thrombin III, activated protein C resistance, and prothrombin G20210A mutation. These thrombophilias cause thrombosis on the venous side of the circulation. However, given our hypothesis of potential transpulmonary shunting of micro-emboli, these parameters may have provided additional information.

## Conclusions

Our case of COVID-19-induced multi-focal large vessel ischemic cerebral infarcts is unique in its excellent outcome and lack of any significant pulmonary disease or manifested coagulopathy. We outline the coagulopathy of COVID-19, contrasting it with the coagulopathy of the Ebola virus. We also echo the recent exciting findings of pulmonary vasodilation and pulmonary shunting of micro-emboli possibly explaining potential embolic infarcts in even mild pulmonary disease. Lastly, we reviewed the recent intriguing neuropathology findings, which do not reveal any significant evidence of a direct vasculopathy. However, our case highlights the possibility of yet undiscovered mechanisms that may underlie the etiology of cerebral ischemic infarcts in seemingly healthy individuals. This case report may prompt us to screen all patients with ischemic strokes for COVID-19 infection. Larger observational studies may dictate guidelines.
